# Persistence of peanut allergen on a table surface

**DOI:** 10.1186/1710-1492-9-7

**Published:** 2013-02-18

**Authors:** Wade TA Watson, AnnMarie Woodrow, Andrew W Stadnyk

**Affiliations:** 1 Department of Pediatrics, Dalhousie University, Halifax, Nova Scotia, Canada; 2 IWK Health Centre, Halifax, Nova Scotia, Canada

**Keywords:** Food allergy, Peanut allergen, Contamination, Ara h 1

## Abstract

**Background:**

A diagnosis of peanut allergy has a major impact on an individual’s quality of life. Exposure to even small amounts of peanut can trigger serious reactions. Common cleaning agents can easily remove peanut allergen from surfaces such as table tops. Parents of children with peanut allergy frequently ask if peanut allergen can persist on surfaces if they have not been cleaned.

**Objectives:**

The purpose of this study was to determine the persistence of peanut allergen on a typical table surface over time.

**Methods:**

Five mL of peanut butter was evenly smeared on a 12 inch by 12 inch (30.5 by 30.5 cm) square on a nonporous (laminated plastic) table surface. Five squares were prepared in the same manner. The table was kept in a regular hospital office at room temperature and ambient lighting. No cleaning occurred for 110 days. Samples were taken at regular intervals from different areas each time. A monoclonal-based ELISA for arachis hypogaea allergen 1 (Ara h 1), range of detection 1.95-2000 ng/mL, was used to assess peanut allergen on the table surface.

**Results:**

At baseline, there was no detectable Ara h 1 allergen. Immediately post application and for 110 days of collecting, detectable Ara h 1 was found each time a sample was taken. There was no obvious allergen degradation over time. Active cleaning of the contaminated surface with a commercial cleaning wipe resulted in no detectable Ara h 1 allergen.

**Conclusions:**

Peanut allergen is very robust. Detectable Ara h 1 was present on the table surface for 110 days. Active cleaning of peanut contaminated surfaces easily removed peanut residue and allergen. Regular cleaning of surfaces before and after eating should be reinforced as a safety measure for all individuals with peanut allergy.

## Introduction

Food allergy affects approximately 4 percent of children under the age of 18 years with a higher prevalence in children less than 5 years [1]. Peanut allergy affects approximately 1.6% of school age children [2]. Peanut is one of the few allergens that is capable of causing life-threatening reactions [3,4]. Current treatment of food allergies includes avoidance of the trigger food and treatment of a severe reaction with epinephrine [5].

The diagnosis of food allergy carries a significant emotional burden on children and their families and affects their quality of life. Mothers of children with food allergies expressed living with fear, worry about well being and looking for control [6]. A diagnosis of food allergy impacted parental perception of general health, parental emotions, and limited family activities [7], with mothers reporting more anxiety and stress compared to fathers [8]. Primeau et al. [9] reported that there was more disruption in family and social interaction in families of children with peanut allergy compared to families with children with a rheumatologic disease. Children with peanut allergy reported poorer quality of life compared with children with insulin dependent diabetes, with more fear reported in managing the peanut allergy than managing the diabetes [10].

Families express major concern about accidental exposure to food in the child’s environment, leading to significant impact for them and the child. A survey of caregivers [11] reported that 60% of participants indicated impact on meal preparation activities, 50% an affect on family social activities and 41% a significant impact on their stress levels due to their child’s food allergy. Ten percent of caregivers did not send their child to school due to food allergy. School activities, such as field trips (59%) and school parties (68%), were significantly affected by food allergy. Sixteen percent of caregivers avoided going to restaurants, 11% avoided allowing their child to play at friends’ houses, 14% avoided daycare or aftercare, 10 to 11% avoided parties and sports, and 26% avoided camp and sleepovers because of the child’s food allergy.

A previous study [12] demonstrated that peanut allergen (Ara h 1) was not widely distributed in preschools and schools. Hand washing and cleaning table surfaces with common cleaning agents easily removed peanut allergen. Guidelines for the management of anaphylaxis in schools and daycare settings have endorsed these recommendations for reducing exposure to allergens [13,14]. While this is reassuring for many families, parents often ask about the risk of exposure to peanut allergen on a surface if the surface has not been cleaned after contamination with peanut. More specifically, how long would peanut allergen persist on a table surface if no cleaning occurred? A review of the medical literature provided no information on the persistence of peanut allergen in the environment.

The purpose of this study was to determine the persistence of peanut allergen (Ara h 1) on a typical table surface if no cleaning occurred.

## Methods

A nonporous table top surface was used (laminated plastic). Five mL of smooth peanut butter was evenly smeared on a 12 by 12 inch (30.5 by 30.5 cm) square. Five squares were prepared in the same manner. The table was kept in a regular hospital office at room temperature and ambient light conditions. Samples for measurement of Ara h 1 were collected prior to the application of peanut butter (baseline), immediately post application (Day 0) and at regular intervals for 110 days (daily for 6 days, twice weekly until Day 28 and at Day 60 and Day 110). Different areas of the table surface were used for each sample. On Day 110, a commercial cleaning wipe, Clorox® Disinfecting Wipes (Clorox Company, Brampton, Ontario, Canada) was used to clean the final square. Another sample for Ara h 1 was collected from the clean surface after the table top air dried, in the same manner as the other samples.

A 37 mm glass fibre filter moistened phosphate buffered saline (PBS) containing 1% Tween 20 was used to sample the table top in a standard fashion. The samples on filters stored at −20 degrees Celsius until extraction. After thawing the filters but prior to the extraction, 1.5 ml of PBS-Tween 20 was added and the samples were left rotating overnight at 4 degrees Celcius. The following day the filters were squeezed to remove all the liquid to fresh tubes, and were tested for the peanut allergen, Ara h 1 by ELISA (INDOOR Biotechnologies, Charlottesville, Va). The samples were diluted 1:5 and 1:50 for testing and the protocol was conducted as provided by the manufacturer. The range of detection of Ara h 1 was between 1.95 and 2000 ng/ml. After analysis the results were multiplied by the dilution factor and expressed as the actual ng/mL for each sample. All samples were assayed at the same time.

## Results

**Figure 1:**
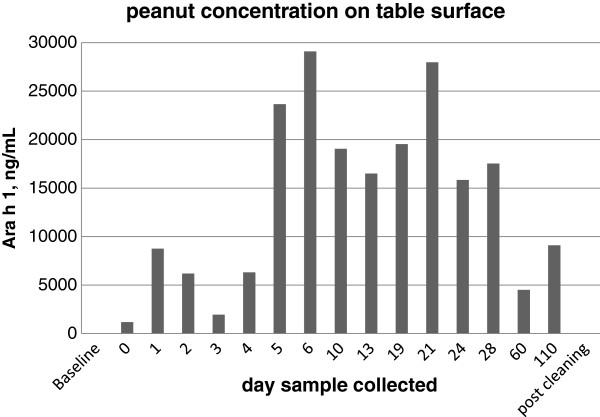
**Concentration of peanut allergen Ara h 1 on a table surface at each collection time. **The table surface was smeared with peanut butter in a standard fashion (5 mL on a 12 by 12 inch square). Samples were collected in a standard fashion.

At baseline, prior to peanut butter application, no detectable Ara h 1 was found on the table surface. Immediately post application, there was detectable Ara h 1 (1184 ng/mL). On every sample collected for 110 days, there was detectable Ara h 1 with ranges of 1951 to 29089 ng/mL. The results are summarized in Figure 1. Immediately after cleaning with the cleaning wipe, no detectable Ara h 1 was found on the table surface.

## Discussion

Peanut allergen is very robust. Detectable Ara h 1 was present on a table surface for 110 days post application. There did not appear to be any allergen degradation over time. Variations in the Ara h 1 levels were likely due to variation of distribution of the peanut butter on the table surface. Active cleaning of the table surface appeared to be the only way of removing the allergen. Even after 110 days, cleaning the surface with a commercial cleaning wipe removed the allergen immediately.

Clinically, this is important information. It reinforces the importance of regular cleaning of surfaces, especially for individuals with a peanut allergy. There are many commercial cleaning wipes, and individuals and families should make it a habit to carry these wipes with them. Simple but thorough cleaning of a surface, for example in a restaurant or a school cafeteria should safely eliminate the peanut allergen.

There are some limitations in this study. After several days the peanut butter was dry on the table surface. The table surface was sampled regularly for peanut allergen using a moistened glass fibre filter. We do not know if touching the contaminated table surface with dry hands would result in significant transfer of peanut allergen. It would be interesting to sample a contaminated surface with no moisture on the filter. We also do not know if other allergens such as milk and egg would persist for this same length of time. These questions could be addressed in a subsequent study.

In conclusion, peanut allergen appears to persist on the table top surface for at least 110 days if no cleaning occurs. The only way to actively remove peanut allergen is by cleaning the surface. Regular cleaning of surfaces before and after eating should be reinforced as a safety measure for all individuals with peanut allergy.

## Competing interests

The authors declare that they have no competing interests.

## Authors’ contributions

WW conceived of the study, participated in the design of the study, collected the data, and drafted the manuscript. AMW participated in the design of the study and assisted in drafting the manuscript. AS carried out the immunoassays and assisted in drafting the manuscript. All authors read and approved the final manuscript.
